# Abdominal wall endometriosis (AWE): Two case reports and literature review

**DOI:** 10.1016/j.ijscr.2023.108495

**Published:** 2023-07-14

**Authors:** Natalia Maria Christina, Valeska Siulinda Candrawinata, Hendry Lie, Kezia Imanuella Godam

**Affiliations:** a Department of Surgery, Faculty of Medicine, Pelita Harapan University, Siloam General Hospital, Tangerang, Indonesia; b Department of Surgery, Faculty of Medicine, Pelita Harapan University, Tangerang, Indonesia; c Faculty of Medicine, Pelita Harapan University, Tangerang, Indonesia

**Keywords:** Abdominal wall endometriosis, Extra pelvic endometriosis, Scar endometriosis, Case report

## Abstract

**Introduction and importance:**

Endometriosis is one of the most frequent gynecologic disorders, clinically confirmed or suspected in one of nine women by the age of 44 years. Its location of occurrence can be classified into intra and extra pelvic. Abdominal wall endometriosis (AWE) is one of its rare location, with frequency of 0.04 to 5.5 %. Furthermore there are only a few cases of AWE in Indonesia that have been reported.

**Case presentation:**

Here we present two Indonesian patients at a General Hospital in Tangerang, Indonesia. The first patient, 26 years old, complained of an umbilical mass 2 years after caesarean section. The second patient, 36 years old, complained of an umbilical mass since 8 months, with no history of prior surgery. Both patients had pre-operative ultrasonography (US) and underwent wide local excision. Histopathology examination with presence of endometrial glandular components and endometrial-like stroma confirmed the diagnosis of AWE.

**Clinical discussion:**

AWE is defined as any endometrial tissue found superficial to the peritoneum, locating most commonly at umbilical, inguinal area, and anterior abdominal wall. Pre-operative diagnostic tools include abdominal ultrasonography (US) or abdominopelvic computed tomography (CT) scan. Since treatment with medications is usually not effective, surgical treatment is recommended, along with confirmation by histopathological examination.

**Conclusion:**

Diagnosis of AWE should be suspected in all women with symptoms of an abdominal mass and cyclic pain, especially if the patient had history of surgery at the abdominal region. AWE is quite rare, but its symptoms can affect quality of life. Hence, a multi-disciplinary approach is necessary, with the strongly recommended treatment of wide local excision to prevent recurrence and malignant transformation.

## Highlights

• Endometriosis is one of the most frequent gynecologic disorders, located at intra or extra-pelvic.
• Abdominal wall endometriosis (AWE) is one of its rare location, which comprises only 0.03 to 0.40% of reported incidence.
• AWE should be suspected in all women with an abdominal mass and cyclic pain, especially with a history of abdominal surgery.
• AWE can affect quality of life and should be treated surgically to prevent recurrence and malignant transformation.

## 1 Introduction

Endometriosis is one of the most frequent gynecologic disorders that is defined as the presence of functioning endometrial glandular epithelium and stroma implants outside of the uterus, accompanied by chronic inflammation [1]. It is clinically confirmed or suspected in 1 of nine women by the age of 44 years [2]. Locating most commonly inside the pelvic cavity, endometriosis can rarely occur outside the pelvic cavity, among which abdominal wall endometriosis (AWE) is the most common extra-pelvic site [3,4]. Endometriosis negatively impacts psychosocial, sexual, and professional aspects as it causes pain, infertility, and other associated dysfunctions, which worsen the quality of life [1].

AWE is defined as any endometrial tissue found superficial to the peritoneum with estimated frequency of 0.04 %–5.5 %. AWE can be classified into primary (spontaneous) and secondary (post-surgical scar) [[5], [6], [7], [8]]. Malignant transformation of AWE is rare with an estimated incidence of 0.3 %–1.0 %, but carries a very poor prognosis [9,10]. These case reports were conducted in line with the SCARE 2020 guideline: updating consensus Surgical Case Report (SCARE) guidelines [11]. Prevalence of AWE is quite rare, even more so in Indonesia population. Here we present two case reports of rare AWE: primary and secondary occurring in Indonesian women.

## 2 Case report 1

A 26-year-old Indonesian woman came to the surgery outpatient department with chief complaint of a painful, palpable, mass of the abdomen since 6 months ago. The patient described that the mass started out as barely noticeable, then enlarging to a diameter of 0.5 cm, and keep enlarging to a diameter of 5 cm on examination. The mass was accompanied by pain that had a cyclic characteristic. During her menstrual period, the pain restricted her daily activities and rated 7 out of 10 on the pain scale. She reported no other abnormalities relating to her menstruation, no fever nor weight loss. The patient had a history of one pregnancy with an uncomplicated caesarean section 2 years prior. The patient denied any hormonal or birth control use. The patient reported no significant personal and family history of any disease, medication and/or genetic condition.

**Fig. 1:**
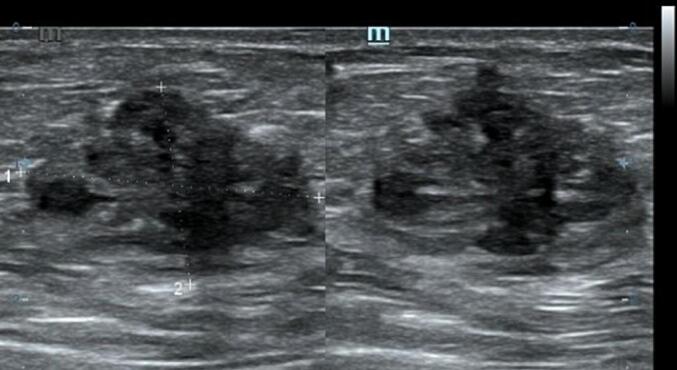
Ultrasonography examination of the first patient showed a heterogenous hypoechoic lesion in the subcutaneous region of the lower abdomen suggestive of endometriosis.

Cutaneous examination of the abdominal region reveals a painful, hard, partially mobile mass about 5 × 5 cm located in the hypogastric region, at the caesarean section scar. A diagnosis abdominal wall endometriosis (AWE) was suspected given the patient's history of caesarean section and flares of pain with menstrual periods. Transabdominal ultrasonography (US) revealed heterogenous hypoechoic lesion in the subcutaneous region of the lower abdomen suggestive of endometriosis (Fig. 1).

**Fig. 2:**
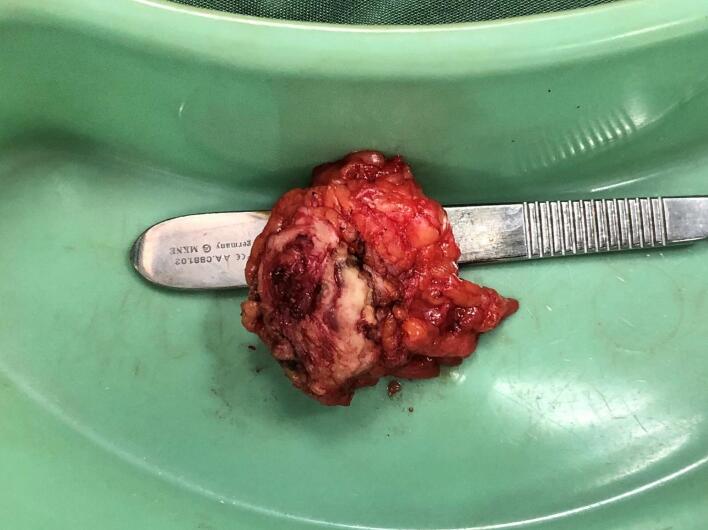
A painful, hard, partially mobile mass about 5 × 5 cm located in the hypogastric region, at the caesarean section scar.

**Fig. 3:**
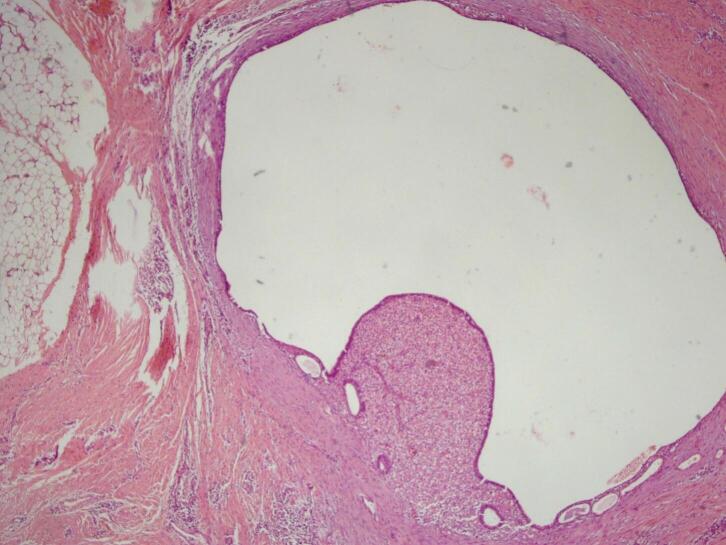
H&E staining, 4× magnification. Endometrial glands and stroma in between fat and fibrotic tissue.

**Fig. 4:**
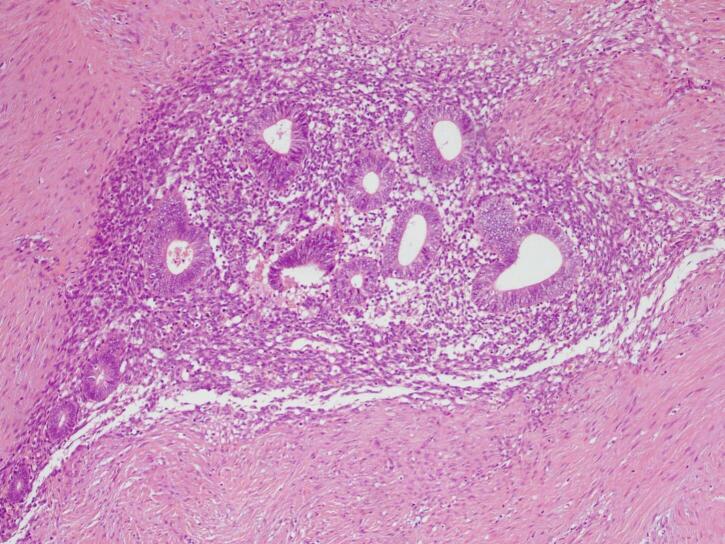
H&E staining, 10× magnification. Endometrial glands and stroma in between fat and fibrotic tissue.

**Fig. 5:**
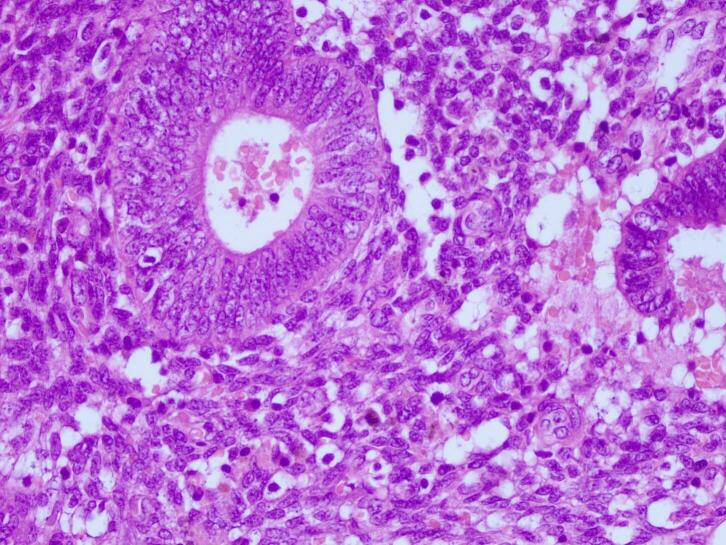
H&E staining, 40× magnification. Endometrial cells were found to be widened, with bleeding and inflammatory, without signs of malignancy.

Surgery of wide local excision with 1 cm free margin was done (Fig. 2), and the histopathology examination result was as follows: endometrial glands and stroma in between fat and fibrotic tissue. Endometrial cells were found to be widened, with bleeding and inflammatory, without signs of malignancy (Fig. 3, Fig. 4, Fig. 5). These findings confirmed the diagnosis of Abdominal wall endometriosis (AWE).

## 3 Case report 2

The second case report is of a 36-year-old Indonesian woman with chief complaint of painful and palpable nodular mass, located in the umbilical region of the abdomen since 8 months ago. The patient also experienced cyclic pain and bleeding related to the onset of her menstrual period. She has no history of menstrual abnormalities or any abdominal or pelvic surgeries. The patient denied any hormonal or birth control use. The patient reported no significant personal and family history of any disease, medication and/or genetic condition.

**Fig. 6:**
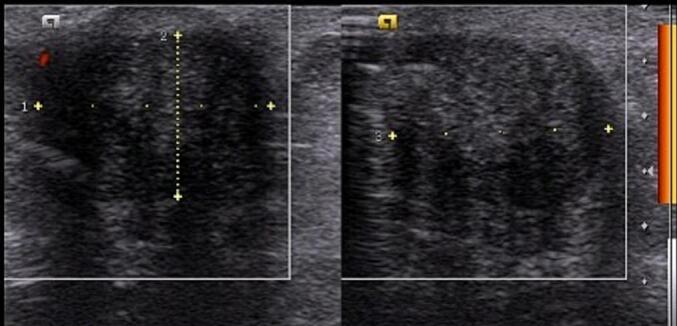
Ultrasonography examination of the second patient showed an inhomogen hypoechoic lesion, hypovascular, 2,12 × 1,46 × 1,96 cm in size with posterior shadowing in the umbilical region of the abdomen.

Cutaneous examination reveals a painful, well-defined, nodular tender mass, 2 × 2 cm in size in the umbilical region of the abdomen. Upon the abdominal ultrasonography (US) examination, we found an inhomogen hypoechoic lesion, hypovascular, 2,12 × 1,46 × 1,96 cm in size with posterior shadowing in the umbilical region of the abdomen suggestive of umbilical endometriosis (Fig. 6).

**Fig. 7:**
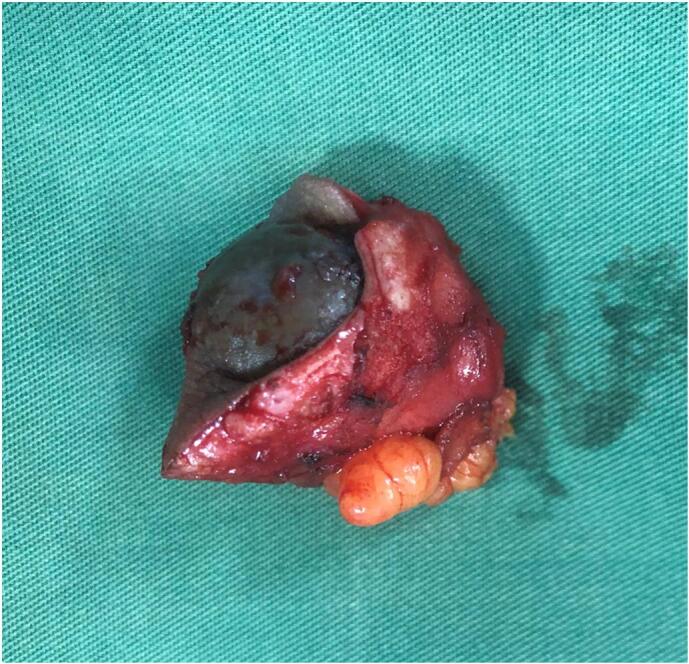
A well-defined, nodular tender mass, 2 × 2 cm in size in the umbilical region of the abdomen.

**Fig. 8:**
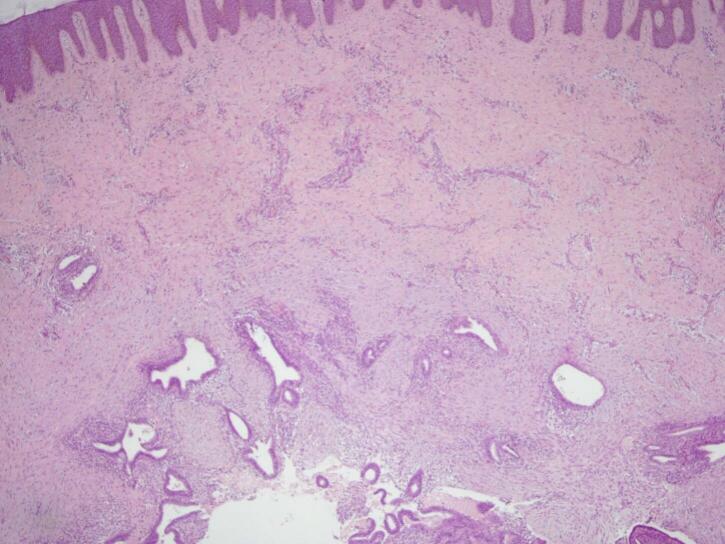
H&E staining, 4× magnification. Endometrial glands lined by cuboid-columnar epithelium.

**Fig. 9:**
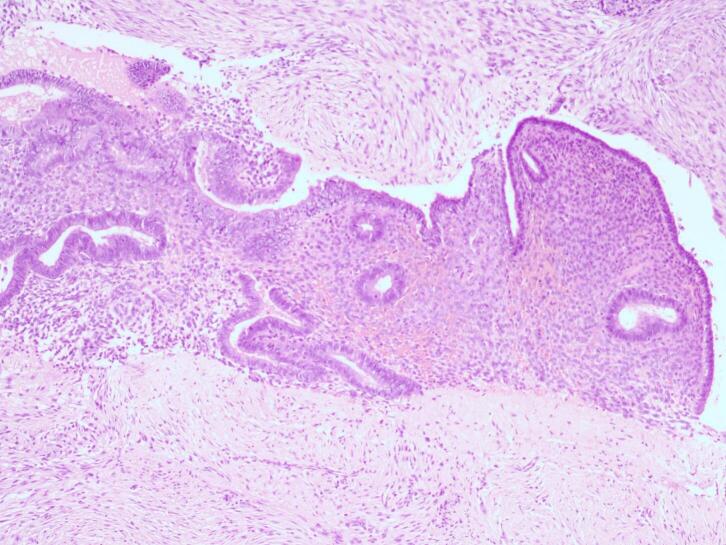
H&E staining, 10× magnification. Endometrial glands and stroma were found in between fibrotic tissue.

**Fig. 10:**
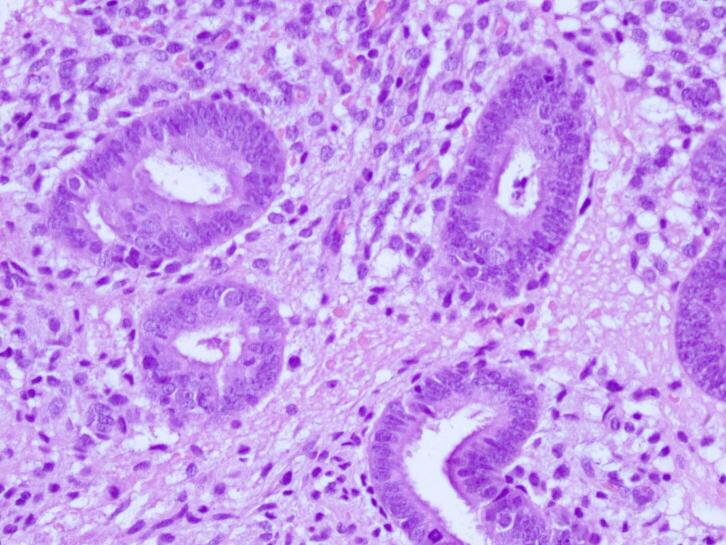
H&E staining, 40× magnification. Endometrial glands and stroma were found in between fibrotic tissue. No signs of malignancy was found.

Treatment of choice in the second patient was also wide local excision with 1 cm free margins (Fig. 7) and histopathology examination was done on the specimens to confirm the diagnosis. Histopathology revealed endometrial glands lined by cuboid-columnar epithelium. Endometrial glands and stroma were found in between fibrotic tissue. No signs of malignancy were found (Fig. 8, Fig. 9, Fig. 10). These findings are compatible with one of AWE types: umbilical endometriosis.

After the surgery, both of the patients stayed in hospital for 1 day and complained of mild pain at excision area. Both patients were discharged with medication of Mefenamic acid 500-mg TID. Follow-up was done 3 and 7 days after surgery at outpatient department with adequate wound healing without any complications.

## 4 Discussion

Endometriosis is one of the most frequent gynecologic disorders that is defined as the presence of functioning endometrial glandular epithelium and stroma implants outside of the uterine [1]. According to a recent 20 years cohort study, one in nine women had clinically confirmed or suspected endometriosis by the age of 44 years, with most diagnosed during their early thirties [2]. Reported incidence of AWE comprises only 0.03 to 0.40 % [2]. Study by Horton et al. found that mean age presentation of AWE is 31 years old, range of age 17–57 years old [5]. A more recent study by Hasan et al. had similar findings with mean age at 35 years, but only included female patients with history of caesarean section [12]. This case series reported two AWE patients with onset of symptoms at the age of 26 and 36 years old, in accordance with the recent prevalence data.

While often located at organs inside the pelvic cavity, endometriosis can also occur outside the pelvic cavity: lung, abdominal wall, or inguinal area [3]. Abdominal Wall Endometriosis (AWE) is defined as any endometrial tissue found superficial to the peritoneum, locating most commonly at umbilical, inguinal area, and anterior abdominal wall [5]. It can be divided into primary when it occurs spontaneously or secondary, which usually develops at the sites of surgical scars, including caesarean section, laparoscopic, hernia repair, and laparotomy [[6], [7], [8]].

A systematic review comprising of 455 AWE patients found that approximately 80 % of the patients had history of prior surgery, with caesarean section being the most common. The mean time interval between surgery and presentation of AWE was 2 to 5 years [5].

The first patient of this case series had secondary AWE at caesarian section incision scar, while the second one had primary AWE as she had no history of prior surgery. Secondary AWE experienced by the first patient had onset of symptoms 2 years after caesarean section.

Presenting symptoms of AWE can include a palpable mass at the abdominal region, characteristic of cyclic pain, general endometriosis symptoms such as dysmenorrhea, dyschezia, or dyspareunia, and/or bleeding from superficial lesion and lower abdominal pain [6]. However, cyclic pain only presents in 57 % of patients, and the most common symptom being mass or pain [5]. Both the patients in this case series experienced a palpable mass with cyclic pain, but the second patient of this case series also experienced bleeding from the lesion.

Pathogenesis of AWE had been a long topic of discussion, among many, there are three that are most influential. Sampson first described theory of implantation, where the endometrial cells reflux and implant on the surrounding pelvic structures during menstruation. This theory was supported with study findings of higher refluxed menstrual blood in women with endometriosis and higher incidence of the disease in females with outflow obstruction. However, it was also found that incidence of retrograde menstruation is similar in women with and without endometriosis leading to the conclusion of a multi-factorial mechanism. Furthermore, this theory does not explain endometriosis occurring outside the pelvic cavity [1,5,13].

The second theory: vascular dissemination is described by Halban where endometrial cells escape from the uterus through lymphovascular channels to the peripheral circulation, then to various sites. The third theory involves metaplasia of the undifferentiated cells in the abdominal wall into endometrial tissue, induced by imitative metaplasia or hormonal manipulation. Other than the tree main theories, immunity is hypothesized to have a bigger role in endometriosis, which might affect the clearance ability of the refluxed endometrial cells. Altered or deficient immunity, more specifically reduced Natural Killer (NK) cells ability and cell-mediated immunity may contribute to the development of endometriosis. Once endometriosis has occurred, a chronic inflammatory process can be observed, evident with the increased number of leukocytes and macrophages. These cells secrete pro-inflammatory cytokines such as IL-6, TNF-α, IL-1 and IL-8 along with vascular endothelial growth factor (VEGF) to increase neoangiogenesis which further contribute to the progression of endometriosis [1,5,13].

A study by Marras et al. suggested that post-surgical AWE is likely caused by iatrogenic implantation of endometrial cells during surgery, while AWE without prior surgery is more likely caused by lymphatic or hematogenous dissemination or metaplasia [14]. Despite many years of research, the exact pathogenesis of AWE remains obscure, but it is highly suspected that the pathogenesis is multi-factorial [1,5,13].

The site of AWE can produce differential diagnosis of hernia, abscess, lipoma, desmoid tumor, hematoma, lymphadenopathy, or malignancy [3,15]. Differentiating AWE from other differentials proves to be challenging, especially if the clinical symptoms are not specific. However, if the hallmark symptoms are present, it might be easier to highly suspect the diagnosis of AWE. Prompt diagnosis might also be important in terms of treatment choices, in which case a patient might choose to undergo hormonal or pharmalogical treatment prior to or as opposed to surgery.

Abdominal ultrasonography (US) or abdominopelvic computed tomography (CT) scan are the most commonly used pre-operative diagnostic tools for suspected AWE [3]. Additionally, magnetic resonance imaging (MRI) and fine-needle aspiration (FNA) are also useful, but the definitive diagnosis can only be confirmed by histopathological examination of the lesion.

Abdominal ultrasonography (US) is the first line and cost efficient diagnostic tool that was used in both of these patients. On ultrasonography, AWE lesion will appear as a heterogenous isoechoic or hypoechoic area surrounded by peripheral vascularization [3,15]. While US remains the best screening tool, CT scan provides better results when endometriosis is located at the muscle or subcutaneous layer and MRI is superior for small lesions [3,15].

On CT scan, the lesion will appear as a solid heterogeneous mass with mild-to-moderate enhancement with administration of intravenous contrast. While on MRI, it will appear as a hyperintense or isointense heterogenous on T1- and T2-weighted images [3,15].

The significant negative impacts of AWE on psychosocial, sexual, and professional aspects demands a multi-disciplinary approach to treat AWE and its possible complications. Effects on quality of life varies from mild to significant restriction of activities depending on severity of pain and/or discomfort. Patient experiencing pain which does not respond to first-line treatment may benefit from a consult with a pain specialist. Gynecologists also have a significant role as hormonal and fertility might also affect sexual aspects of the patient. Infertility might be the result of pelvic adhesions, impairment of oocyte release or pick-up, disorder of myometrial contractions, and impairment of fertilization and embryo transport [1,13].

There have been multiple studies concerning the efficacy of pharmalogical treatments with ambiguous results. Pharmacological treatment can be initiated on the basis of clinical suspicion and has the benefits of symptoms relief and inhibition of further development of endometriosis. However, pharmacological treatments have shown no improvement in endometriosis-associated infertility and generally should be discouraged in patients who desire a live birth, except those undergoing in-vitro fertilization (IVF) [13].

Administration of drugs such as non-steroidal anti-inflammatory drugs (NSAIDs), contraceptives, gonadotropin-releasing hormone (GnRH) agonists and aromatase inhibitors showed improvement of symptoms, but no change in lesion size. Discontinuation of treatment is also likely to cause recurrence of symptoms [10,13,16,17].

Pharmalogical treatment might serve to eliminate symptoms prior to surgery or as a treatment of choice in patients who refused to undergo surgery [1]. Wide excision remains the mainstay and only definitive treatment of AWE to achieve complete excision, alleviation of symptoms and prevention of malignant transformation [10,[13], [14], [15], [16], [17]]. In both our cases, the patient was not given any pharmacological treatment as they both wished to have more children and agreed to undergo surgery of wide excision.

There is currently no recommendation or mutual agreement on how wide the negative margins should be, but there had been suggestion of 1 cm minimal margins to prevent recurrence and/or malignant transformation (occurring in 1 % of endometriosis) [14,16]. In both patients, a wide local excision with 1 cm margins was done and the specimens were sent to histopathology examination for definitive diagnosis.

Two histological features out of three diagnostic triads are required to diagnose endometriosis: endometrial glands, endometrial stroma or hemosiderin pigments [18]. Histopathology examination in both patients showed both endometrial glands and endometrial stroma, without any signs of malignancy. Hence, the diagnosis of AWE were confirmed in both patients.

Recurrence rate after wide local excision is much lower (1.5 %–7.5 %) when compared to medical treatment [15,19]. As previously mentioned, endometriosis at any location have 1 % risk of related-malignant transformation, most often being clear-cell carcinoma, which has good outcomes and 80 % 5-year survival rate [19].

AWE case reports in the Indonesian population are still limited, but it might be under-reported. Diagnostic and treatment guidelines of AWE would be beneficial as this disease implicate several important aspects (restriction of activities, psychosocial, sexual, and professional aspects) which can significantly decrease quality of life.

## 5 Conclusion

Diagnosis of AWE should be suspected in all women with symptoms of an abdominal mass and cyclic pain, especially if the patient had history of surgery at the abdominal region. AWE is quite rare, but its symptoms can affect quality of life. Hence, a multi-disciplinary approach is necessary, with the strongly recommended treatment of wide local excision to prevent recurrence and malignant transformation.

## Patient perspective

At 1-day post-op follow up, both patients felt relieved and content that the tumor had been excised. At 3-days post-op follow up, the first patient still complained of mild pain, but at 7-days post-op follow up, she was content that the pain had decreased and the scar resulted was healing well. The second patient experienced minimal pain and expressed satisfaction at the scar result at 3-days and 7-days post-op follow up.

## Consent

Written informed consent was obtained from patients about these case reports writing and publishing. Both patients understood well and gave consent. A copy of the written consent is available for review by the Editor-in-Chief of this journal on request.

## Ethical approval

As it is a case report, ethical approval is exempted by our institution.

## Funding

This research received no specific grant from any funding agency in the public, commercial, or not-for-profit sectors.

## Author contribution

NMC, VS, HL, KIG: the conception and design of the study, or acquisition of data, or analysis and interpretation of data, drafting the article or revising it critically for important intellectual content, final approval of the version to be submitted.

## Guarantor

Natalia Maria Christina acts as the guarantor of this study.

## Declaration of competing interest

The authors declare that there is no conflict of interest.
